# Oral Health Status of Children Living with Type 1 Diabetes Mellitus

**DOI:** 10.3390/ijerph19010545

**Published:** 2022-01-04

**Authors:** Dorottya Banyai, Daniel Vegh, Adam Vegh, Marta Ujpal, Michael Payer, Zita Biczo, Zsuzsanna Triebl, Khaled Mukaddam, Valentin Herber, Norbert Jakse, Zsolt Nemeth, Peter Hermann, Noémi Rózsa

**Affiliations:** 1 Department of Paediatric Dentistry and Orthodontics, Semmelweis University, 1088 Budapest, Hungary; dorottya.banyai.official@gmail.com (D.B.); rozsa.noemi@dent.semmelweis-univ.hu (N.R.); 2 Diabetes-Dental Working Group, Semmelweis University, 1088 Budapest, Hungary; adam.vegh.92@gmail.com (A.V.); martaujpal@gmail.com (M.U.); zita.biczo@gmail.com (Z.B.); zstriebl@gmail.com (Z.T.); nemeth.zsolt@dent.semmelweis-univ.hu (Z.N.); hermann.peter@dent.semmelweis-univ.hu (P.H.); 3 Department of Prosthodontics, Semmelweis University, 1088 Budapest, Hungary; 4 Department of Dentistry and Oral Health, Division of Oral Surgery and Orthodontics, Medical University of Graz, 8010 Graz, Austria; mi.payer@medunigraz.at (M.P.); valentin.herber@medunigraz.at (V.H.); norbert.jakse@medunigraz.at (N.J.); 5 Department of Maxillofacial and Oral Surgery, Semmelweis University, 1088 Budapest, Hungary; 6 Faculty of Dentistry, Semmelweis University, 1088 Budapest, Hungary; 7 University Center for Dental Medicine, Department of Oral Surgery, University of Basel, 4058 Basel, Switzerland; khaled.mukaddam@unibas.ch

**Keywords:** type 1 diabetes mellitus, orthodontics, diabetes mellitus, oral health, coeliac disease

## Abstract

Background: Diabetes is a well-known predisposing factor for oral diseases, so prevention in an early age is mandatory. Objective: To provide oral screening for children living with type 1 diabetes. We aimed to investigate the oral and general health indexes of T1DM children and compare these data to healthy siblings and controls. Methods: In this cross-sectional study, 120 DM patients and 78 siblings, thereafter 80 DM children and 95 controls, took part. A detailed questionnaire, panoramic radiographs, and lateral cephalograms were obtained in every orthodontic consultation. We used Pearson’s chi-square test for statistical analysis and compared the data of the study and control groups. Results: The oral health values of DM children were significantly better (DMF-T 0.83–1.3) than the national average (3.8–4.5). A total of 75% (*n* = 60) of the children needed orthodontic treatment for orthodontic or skeletal anomalies. The prevalence of skeletal anomalies was significantly (*p* < 0.05) higher among patients with diabetes mellitus (DM) than in the control group. The frequency of coeliac disease was significantly elevated compared to any literature data (1–3.5%) in the study (15%) and in the control sibling group (13%). Conclusions: Co-morbidities such as CD should get more attention as a prognostic factor for a future higher incidence of diabetes. T1DM children can be motivated and health-conscious patients with excellent oral hygiene and dental status. Orthodontic treatment can help eliminate the oral complications of DM. Special diabetes ambulances may help provide oral care for patients with DM.

## 1. Introduction

Diabetes mellitus (DM) is a chronic illness that affects 420 million people worldwide and approximately 700,000 people in Hungary. In 2019, more than 600,000 children under the age of 15 had diabetes worldwide, and 1 in every 600 children had type 1 diabetes in Hungary [1,2,3]. Approximately 90% of affected patients have type 2 DM (T2DM), and 10% have T1DM.

T1DM is treated with intensive insulin therapy. Optimal timing of treatment can reduce microvascular and macrovascular complications [4,5]. The international guidelines are not strict, but individualised treatment is recommended. For example, in people receiving intensive insulin therapy and those with newly diagnosed T2DM, hemoglobin A1C (HbA1c) should be reduced to <7.0%; however, 6% and 8% have also been recommended in position statements and guidelines [4,5,6].

Many people living with DM suffer from anxiety, depression, and other mental health problems. In addition, a diagnosis of DM in early childhood can negatively affect the patient’s emotional development [7,8].

Oral health education is essential because DM can harm dietary habits [2,9]. Unplanned extra carbohydrate consumption and sugary drinks at night can result from hypoglycemia, interfering with proper oral hygiene. Hyperglycemia may lead to xerostomia, decreasing the buffering effects of saliva for the dentition and leading to caries. Oral infections, such as candidiasis, are well-known factors in the development of caries. The Diabetes Dental Working Group provides free oral screening services to reduce the prevalence of childhood caries.

Technological advances in the treatment of DM and the availability of affordable and high-quality insulin analogs have enabled proper metabolic control and increased the life expectancy of patients living with T1DM to be in line with those who do not have DM [10]. In patients with DM, regular medical and dental check-ups can help prevent complications [11,12]. The disease is not always easily manageable, but in patients with an early diagnosis of DM, life expectancy can be 65 years or more throughout the world [13].

Inflammation adversely affects metabolic levels; inflammation in the oral cavity or elsewhere in the body can delay wound healing, but insulin or medication adjustments can help maintain a healthy blood glucose level [14]. Dental treatment can help prevent dental decay and oral inflammation. Orthodontic treatment aims to make surfaces more accessible for cleaning. In children, orthodontists must help with education and treatment before the onset of dental disease results from a lack of motivation or successful cleaning. Through orthodontic consultation and treatment for young people living with T1DM, we believe we can help the community by making care more accessible, and we work closely with the Hungarian Diabetes Association and International Diabetes Federation to keep diabetologists up to date about this service.

In Hungary, free dental care is provided for all patients younger than 18. Our service is not limited to location; it is available to every child in Hungary living with T1DM.

The university provides the background and human resources for examination and treatment, and all university-based departments can refer patients to us if necessary. By studying whether DM affects oral health and skeletal growth, we aim to share our knowledge and experiences with international colleagues and motivate other countries to provide the same service if possible. Many previous studies focused on the correlation between early-onset DM and skeletal growth anomalies, but the results were not noticeable [2,15,16,17,18,19,20,21,22]. It is well-known that the growth hormone (GH)/insulin-like growth factor-1 (IGF-1) axis which regulates growth is affected by T1DM, with studies showing increased GH and decreased IGF-1 levels in children with T1DM. This can affect craniofacial development with a higher incidence of dysgnathic patterns in children with T1DM [16,23].

## 2. Methods

### 2.1. Patients

We organised four free oral screenings for people with DM in the World Diabetes Day events supported by the biggest adult and pediatric diabetes foundations in 2018 and 2019. These events were attended by hundreds of children with T1DM. Since the outbreak of the coronavirus disease 2019 (COVID-19) pandemic, however, on-site patient events have been unavailable to the public. Therefore, we continued our study, the so-called ‘Budapest pilot’, in a more controlled environment at the Department of Paediatric Dentistry and Orthodontics, Semmelweis University. We provided university-based free oral screening for people with T1DM. In addition, we shared information about screening on social media via DM non-governmental organizations (NGOs), DM associations, and DM foundations. The study was approved by the Semmelweis University Ethical Board (RKEB: 204/2018) and was conducted following the Declaration of Helsinki.

### 2.2. Data Screening by the On-Site Patient Event

We analysed the data of 120 children younger than 18 years living with DM and 78 healthy siblings as a control group. Each patient or a parent filled out a questionnaire about basic medical information, such as age, existing illness, and allergies.

The next step was to examine patients’ oral health status (oral hygiene index simplified (OHI-S) and decayed, missing, or filled teeth (DMF-T)) and the existence of dental or skeletal orthodontic anomalies. Then, we looked for significant differences between the study group and the control group. Finally, we also examined the type and frequency of insulin treatment.

The examination event was held twice in 2018 and twice in 2019.

### 2.3. Data Screening by the Diabetes Ambulance (Department of Paediatric Dentistry and Orthodontics)

We collected the data of 80 children younger than 18 years old who were living with DM. In addition to the oral health indicators (OHI-S and DMF-T), we documented general health issues such as coexisting chronic illnesses, the type of DM therapy, and any skeletal orthodontic anomalies. After children and parents filled out the questionnaire and after the oral health examination, lateral cephalograms were also obtained and analysed through the Ricketts and Hasund methods with OnyxCeph dental imaging software (Image Instruments, Chemnitz, Germany). All the linear and angular measurements were performed by two experienced orthodontists. We used the average of the two measurements when their discrepancy was within 0.5 mm for the linear or 0.5 for angular values.

In addition, HbA1c values were measured with a point-of-care device (SmartTester, 77 Elektronika Kft., Budapest, Hungary). SmartTester is a rapid test reader for in vitro diagnostic use designed to evaluate the related lateral flow tests with image-processing-based measurement technology.

Finally, we compared the cephalogram values of these 80 children with those of 95 control patients. 

The examination period was from 1 September 2020 to 30 August 2021.

### 2.4. Statistical Method

We used Prism 8.4.2 software (GraphPad Software, San Diego, CA, USA) to evaluate the data analysis, calculated as the means ± standard deviations and ranges or as absolute numbers with percentages. We used Pearson’s chi-square test for statistical analysis. Differences with a *p*-value of <0.05 were considered significant. For visualization, we used Tableau Public (Tableau Software, Seattle, WA, USA).

## 3. Results

### 3.1. On-Site Patient Events

Before the COVID-19 pandemic, the Diabetes Dental Research Group provided free oral screenings for people living with DM at public events. In 2018 and 2019, we collected the data of 120 children with T1DM at on-site patient events. We compared the data of 120 children with T1DM with those of their siblings (*n* = 78) in terms of general and oral health and the frequency of skeletal orthodontic anomalies (Table 1) and found that skeletal orthodontic anomalies were far more common among the children with T1DM (92%) than among the sibling controls (76%). We also examined the metabolic parameters and the types of insulin therapy of children living with T1DM.

The DMF-T values of both the children with T1DM and their siblings (DMF-T < 3.5) were correlated with general European values. However, the value of DMF-T was lower than 3 in 88% of patients with T1DM (*n* = 106) and 92.3% (*n* = 72) of the controls. Of the children with T1DM, 71.7% (*n* = 86) had orthodontic anomalies and OHI-S values 1 to 3. Of the controls, 64% (*n* = 50) had orthodontic anomalies.

Of the children with T1DM, 52% (*n* = 62) used injection pens; of those 62 patients, 6% (*n* = 4) also received continuous glucose monitoring (CGM), and the other 94% (*n* = 58) received only human recombinant insulin (Humulin R). A total of 48% of the patients (*n* = 58) received insulin pump therapy, and of those, 47% (*n* = 27) also received CGM. The mean OHI-S value of pump therapy patients was 0.4.

Compared with the healthy general population, the frequency of coeliac disease among the patients with T1DM (15%) and siblings (13%) was significantly higher than in any previous reports (3.5% for DM and 1–2% for average children) [24,25,26,27]. Coeliac disease often coexists with T1DM, and the presence of one may predispose patients to the development of the other [25].

### 3.2. Diabetes Ambulance (Department of Paediatric Dentistry and Orthodontics)

In previous studies, correlations between early-onset T1DM and skeletal growth were investigated [15,16,17,18,19,20,21,22,23]. Therefore, we examined the severity of orthodontic skeletal anomalies in 80 children with T1DM and 95 controls who did not have DM. The control group consisted of children randomly chosen from patients treated at the Department of Paediatric Dentistry and Orthodontics between 2019 and 2020. The exclusion criteria were the existence of any genetic or metabolic disorders. We used the Hasund and Ricketts methods to analyse the lateral cephalograms with OnyxCeph software and compared the severity of the discrepancies in the patients with DM and the controls.

Of the 80 patients with T1DM, 55% (*n* = 44) were male, and 45% (*n* = 36) were female. The mean age was 10.4 years (range 4 to 19 years). At the examination, the mean duration of T1DM was 4.4 years (range three months to 13 years). A total of 32.5% of the patients (*n* = 26) had biopsy-proven coeliac disease, 5% (*n* = 4) had lactose intolerance and 2.5% (*n* = 2) had thyroid disease. Furthermore, 10% (*n* = 8) had siblings with coeliac disease, and 7.5% (*n* = 6) had siblings with DM. In one set of triplets, all 3 had coeliac disease, and 2 had T1DM.

We also examined some metabolic parameters (Table 2). The mean blood glucose level was 8.36 mmol/L (range 3.8 to 20 mmol/L), and the HbA1c level was 6.96% (range 6% to 12%). The mean ketone level was 0.13 (range 0.1 to 0.2). HbA1c values were lower than 8 in 87.5% of the patients (*n* = 70) and lower than 7 in 52.5% of the cases (*n* = 42).

Our on-site study results (Table 3) revealed discrepancies in the oral health status between the children using pump therapy and those using pens and even among children with blood glucose sensors. Of the patients with DM, 57.5% (*n* = 46) used pump therapy and CGM. The mean duration of this type of treatment was 3.6 years (range 1 to 9 years). Of the children using pump therapy, 52% (*n* = 24) used insulin aspart (NovoRapid), 4.3% (*n* = 2) used regular insulin (Actrapid), and 43.5% (*n* = 20) used insulin lispro (Humalog).

Of the patients with T1DM, 42.5% (*n* = 34) received pen treatment, and 70.6% (*n* = 24) also used sensors. Humulin R, Actrapid, and insulin degludec (Tresiba) are the types of insulin most commonly used in pen therapy. A short-acting insulin formulation (Humulin R) was used by 41.2% (*n* = 14); Actrapid by 35.3% (*n* = 12), and a long-acting insulin formulation (Tresiba) by 29.5% (*n* = 12).

Of the 70 patients using glucose monitoring systems, 98.5% (*n* = 69) used Guardian Connect sensors (Medtronic, Dublin, Ireland), and 1 used a FreeStyle Libre system (Abbott Laboratories, Chicago, IL, USA). Of the 80 patients with T1DM, 67.5% (*n* = 54) had mixed dentition, 20% (*n* = 16) had only permanent dentition, and 12.5% (*n* = 10) had only primary dentition.

OHI-S values were ≤1 in 85% of T1DM patients (*n* = 68) and DMF-T values were ≤1 in 71.3% (*n* = 57). In a previous study, the Hungarian average DMF-T values were 4.5 at 5 to 6 years old and 3.8 in patients under 12 years old [28].

Of the children with T1DM, 75% (*n* = 60) had visibly recognizable severe orthodontic anomalies. We analysed the lateral cephalograms of these 80 children (Table 4) mainly using the Ricketts and Hasund methods. In addition, we compared the degree of the discrepancies between those patients and the control group. The control group consisted of 95 children whose mean age was 13.5 years (range 7 to 18); 64.2% (*n* = 61) were female, and 35.8% (*n* = 34) were male.

We examined the discrepancies in typical cephalometric values, such as 5% or 10% decreases and increases. Among the children with T1DM, a decline in the maxillary prognathism (SNA) (26%) and the mandibular prognathism (SNB) values (30%) were most common, whereas among the controls, elevated SNA (17.9%) and decreased SNB values (27.4%) were frequent.

The inclination of the mandible (NL-ML) was decreased in 70% of the patients with T1DM and 57.9% of the controls. In contrast, an inclination of the maxilla (NL-NSL) was increased in both the control and study groups (70.5% and 77.5%). We also found differences in interbasal relationship (ML-NSL) values: Among the patients with T1DM, a decrease was more common (47.5%), whereas, among the controls, an increase was more common (47.4%). In addition, the gonion angle was decreased in both the patients with T1DM (33.8%) and the controls (20%).

The Wits values were lower than 0 mm in 45% of the patients with T1DM and the normal range in 25%. Those values were lower in 34.7% of the controls and in the normal range for 39%.

The ratio of posterior facial height to anterior facial height (PFH/AFH) was increased in 80% of patients with T1DM and 55.8% of the controls. However, many patients (41.3%) had reduced lower facial height, whereas most controls (72.6%) had average values.

The interincisal angle was decreased in 7.8% of patients with T1DM, and it was in the normal range in 51.6% of the controls. The incisor mandibular plane angle (IMPA) values were mainly elevated in the patients (55%) and many controls (40%), but in 44.2% of the controls, they were in the normal range. The facial axis and *Y* axis were in the average range in both groups.

## 4. Discussion

Our study is a borderline topic from the aspect of a diabetologist; however, it contains evidence as to why interdisciplinary cooperation is crucial between dentists and diabetologists.

It is essential to conduct a dental examination and probably an orthodontic consultation for every newly diagnosed child with T1DM. In addition, we found that skeletal differences were significant in the T1DM group.

After successful orthodontic treatment, it is easier to maintain proper oral hygiene if the teeth are well aligned, as the brushing becomes manageable. As T1DM is a risk factor for oral complications, the aim is to create accessible standards and straightforward dental care advice for our colleagues.

Moreover, it is advised to involve oral screening in the diabetes care protocol and make it possible to refer our patients to a dentist or orthodontist at an early age of the diagnosis. At this age, it is possible to provide less invasive treatment. Additionally, orthodontic treatment evaluation is most accessible due to the excellent level of bone remodeling capability.

The oral health indicators, such as DMF-T values, of the patients with T1DM in both the on-site (mean 0.83) and university screenings (mean 1.3) were significantly better than the Hungarian average of 3.8 to 4.5. In the on-site screening, a large majority of the patients had DMF-T values of ≤1, and in the university screening, a majority had DMF-T values of ≤1. Coeliac disease was far more common among the patients in on-site (15%) and university screenings (22.5%) than in the literature data (3.5%). On-site screening results showed that some patients had both DM and coeliac disease, whereas the university screening group had more patients with both conditions.

Skeletal orthodontic anomalies were present in almost all the children with coexisting orthodontic anomalies and T1DM (92% of all orthodontic anomalies were skeletal abnormalities) but far fewer in sibling controls (76% had skeletal discrepancies). It is interesting to consider whether genetic factors may or may not have played a role in this finding.

In general, lateral cephalogram values that were discrepant from average values and ranges were more common in the DM study group than in the control group. The majority of controls had values in the normal ranges. Discrepancies from normal SNA, NL-ML, and ML-NSL values and their prevalence in the DM group were significantly higher than the frequency of the same anomalies in the control group. In addition, PFH/AFH ratios, lower facial height, IMPA, and interincisal angle values deviated significantly more from normal ranges in the study IDDM group than among the control cases.

DM affects the facial structure and skeletal maturation, mainly when metabolic disorders manifest before prepubertal growth spurs, especially in early-onset cases. The mean duration of DM and the mean age in our study correspond to the data in previous studies [15].

Patients who had pump therapy had better oral hygiene parameters than did those who used pens. Thus, compliance with the regimen of pump therapy must be stringent, which may explain why these children had proper oral hygiene.

Orthodontic treatment is not contraindicated in patients with well-controlled DM. Poor metabolic control must consider the advantages and disadvantages of orthodontic treatment, worsening oral hygiene, and reducing bone remodeling capacity [29,30,31]. Because the level of health consciousness may be high in children who receive pump therapy and CGM, their quality of life may be better, and the chance for DM-related healthcare complications may be lower [32].

It is necessary to highlight the importance of oral screening and oral health education for people living with DM. This kind of care is provided in Hungary through oral screening and university-based orthodontic diagnostic and treatment opportunities. In children living with T1DM, oral health and oral care are core points in overall DM care. In addition, well-aligned and easily brushable teeth are more important in patients who live with DM: hypoglycemia occasionally results from intensive insulin therapy, and in this scenario, patients need to consume fast-absorbing carbohydrates, sugary drinks, or sugar. These periods can occur during the night when cleaning the teeth after consumption may be difficult.

Every dental and skeletal abnormality is a risk factor for caries, tooth loss, or periodontitis. Orthodontic treatment can help eliminate these complications by providing opportunities to clean the oral cavity and teeth. The optimal number and quality of teeth are essential; however, optimal position and occlusion are also mandatory for nutrition and cleaning.

We hope to investigate further the benefits of orthodontic care in children with T1DM; evidence about the financial aspects of such care can be a foundation for epidemiological and dental programs worldwide. The Diabetes Dental Working Group aims to improve the oral health of patients with DM with the help of such programs and projects, which would support the need for regular dental visits. As we understand from our experiences, dental and medical societies consider oral health essential for overall health.

## 5. Conclusions

Co-morbidities such as CD should get more attention as a prognostic factor for a future higher incidence of diabetes. Children with T1DM can be motivated and health-conscious patients with excellent oral hygiene and dental status. Orthodontic treatment can help eliminate the oral complications of DM. Special diabetes ambulances and proper education may help provide oral care for patients with DM. We advise annual dental check-ups and orthodontic consultation for every child diagnosed with T1DM. Our dental screening activity (called Budapest pilot) is a great initiative to strengthen interdisciplinary cooperation and help patients with chronic metabolic disorders.

## Figures and Tables

**Table 1:** On-site screening data evaluation.

Characteristic		Type 1 Diabetes Mellitus (*n* = 120)	Control (*n* = 78)	*p* Value *
Gender	Female	68	48	0.496493
	Male	52	30	
Mean age		8 (range 3 to 18)	8 (range 3 to 18)	
Anomalies	Orthodontic	72	50	0.494576
	Skeletal	66	38	
OHI-S	0	58	50	0.012765 *
	1	46	22	
	2	18	4	
	3	0	2	
	Mean	0.7	0.5	
DMF-T index	0	92	50	0.054897
	1	6	14	
	2	4	6	
	3	4	2	
	4	4	2	
	5	2	0	
	6	8	4	
	Mean	0.83	0.63	
Therapy type	Pen	62	n.a.	
	Pump	58	n.a.	
Frequency of coeliac disease	This study	18 (15%)	10 (13%)	0.031083 *
	Other literature	3.5%	1% to 2%	

DMF-T, decayed, missing, or filled teeth; n.a., not available; OHI-S, oral hygiene index simplified. * Boldface indicates significance. (* *p* < 0.05).

**Table 2:** Metabolic parameters of the university-based data evaluation.

Parameter	Patients with Type 1 Diabetes Mellitus (*n* = 80)
Mean blood glucose level	8.36 mmol/L (range 3.8 to 20 mmol/L)
Mean HbA1C level	6.96% (range 6% to 12%)
Ketone value	0.13 (0.1 to 0.2)

HbA1C, haemoglobin A1C.

**Table 3:** Screening evaluation data were obtained from the Department of Paediatric Dentistry and Orthodontics diabetes ambulance and control group.

Characteristic		Type 1 Diabetes Mellitus (*n* = 80)	Control (*n* = 95)	*p* Value *
Gender	Female	36	61	0.010864 *
	Male	44	34	
Mean age		10.4 (range 5 to 18)	13.5 (range 7 to 18)	
Anomalies	Orthodontic	60	95	0.017086 *
	Skeletal	46	38	
OHI-S	0	32	44	0.587905
	1	36	40	
	2	4	6	
	3	8	5	
	Mean	0.85	0.71	
DMF-T index	0	53	67	0.074063
	1	4	14	
	2	6	5	
	3	6	3	
	4 or more	11	6	
	Mean	1.3	0.6	
Therapy type	pen	62	n.a.	n.a.
	pump	58	n.a.	
Frequency of coeliac disease	This study	18 (22.5%)	0 ^†^	
	Other literature	3.5%		

DMF-T, decayed, missing, or filled teeth; n.a., not available; OHI-S, oral hygiene index simplified. * Boldface indicates significance. ^†^ This was an exclusion criterion.

**Table 4:** Lateral cephalometric values and evaluation data from the Department of Paediatric Dentistry and Orthodontics diabetes ambulance and control group.

**Normal Range/Value**	**Type 1 Diabetes Mellitus (*n* = 80)**				**Control (*n* = 95)**				***p*** Value *
	**Lower than Average Range/Value**		**Higher than Average Range/Value**		**Lower than Average Range/Value**		**Higher than Average Range/Value**		
Cephalometric value (normal)	>5% decrease	>10% decrease	>5% increase	>10% increase	>5% decrease	>10% decrease	>5% increase	>10% increase	
SNA (82°)	21 (26%)	14 (17.5%)	12 (15%)	0	7 (7.4%)	1 (1.1%)	17 (17.9%)	2 (2.1%)	**0.000719**
SNB (80°)	24 (30%)	6 (7.5%)	3 (3.8%)	0	26 (27.4%)	5 (5.3%)	7 (7.4%)	3 (3.2%)	0.266668 (n.s.)
NL-ML (23.5°)	56 (70%)	50 (62.5%)	12 (15%)	8 (10%)	55 (57.9%)	40 (42.1%)	31 (32.6%)	23 (24.2%)	**0.001856**
NL-NSL (8.5°)	12 (15%)	8 (10%)	62 (77.5%)	59 (73.8%)	18 (18.9%)	16 (16.8%)	67 (70.5%)	62 (65.2%)	0.465233 (n.s.)
ML-NSL (32°)	38 (47.5%)	30 (37.5%)	30 (37.5%)	12 (15%)	33 (34.7%)	23 (24.2%)	45 (47.4%)	40 (42.1%)	**0.001267**
Gonion angle (126°)	27 (33.8%)	3 (3.8%)	12 (15%)	0	19 (20%)	6 (6.3%)	15 (15.8%)	2 (2.1%)	0.193107 (n.s.)
**Normal Range/Value**	**Type 1 Diabetes Mellitus (*n* = 80)**				**Control (*n* = 95)**				***p*** Value *
**Cephalometric Value (Normal)**	**Lower than Normal Value/Range**		**Higher than Normal Value/Range**	**Normal**	**Lower than Normal Value/Range**	**Higher than Normal Value/Range**	**Normal**		
Wits value (0 to 2 mm)	36 (45%)		24 (30%)	20 (25%)	33 (34.7%)	25 (26.3%)	37 (38.9%)		0.137764 (n.s.)
PFH/AFH (59% to 63%)	6 (7.5%)		64 (80%)	10 (12.5%)	6 (6.3%)	53 (55.8%)	36 (37.9%)		**0.000692**
Lower FH (45° ± 4°)	33 (41.3%)		6 (7.5%)	41 (51.3%)	11 (11.6%)	15 (15.8%)	69 (72.6%)		**0.000029**
Interincisal angle (130° to 150°)	59 (73.8%)		6 (7.5%)	15 (18.8%)	40 (42.1%)	6 (6.3%)	49 (51.6%)		**0.000034**
IMPA (90° ± 5°)	3 (3.8%)		44 (55%)	33 (41.3%)	15 (15.8%)	38 (40%)	42 (44.2%)		**0.015809**
Facial axis (90° ± 3.5°)	16 (20%)		16 (20%)	48 (60%)	23 (24.2%)	15 (15.8%)	57 (60%)		0.677018 (n.s.)
*Y* axis (53° to 66°)	0		0	80 (100%)	5 (5.3%)	0	90 (94.7%)		

AFH, anterior facial height; DM, diabetes mellitus; FH, Frankfort horizontal plane; IMPA, incisor mandibular plane angle; ML, mandibular jaw baseline; NL, maxillary jaw baseline; n.s., nonsignificant; NSL, anterior cranial baseline; PFH, posterior facial height; SNA, sella–nasion–A point angle; SNB, sella–nasion–B point angle. * Boldface indicates significance.

## Data Availability

The datasets used and/or analyzed during the current study are available from the corresponding author on reasonable request.
